# Reporters for Single-Cell Analysis of Colicin Ib Expression in *Salmonella enterica* Serovar Typhimurium

**DOI:** 10.1371/journal.pone.0144647

**Published:** 2015-12-10

**Authors:** Stefanie Spriewald, Jana Glaser, Markus Beutler, Martin B. Koeppel, Bärbel Stecher

**Affiliations:** 1 Max-von-Pettenkofer Institute, LMU Munich, Pettenkoferstr. 9a, 80336 Munich, Germany; 2 German Centre for Infection Research (DZIF), partner site LMU Munich, Munich Germany; Centre National de la Recherche Scientifique, Aix-Marseille Université, FRANCE

## Abstract

Colicins are toxins that mediate interference competition in microbial ecosystems. They serve as a “common good” for the entire producer population but are synthesized by only few members which pay the costs of colicin production. We have previously shown that production of colicin Ib (*cib*), a group B colicin, confers a competitive advantage to *Salmonella enterica* serovar Typhimurium (*S*. Tm) over commensal *E*. *coli* strains. Here, we studied regulation of *S*. Tm *cib* expression at the single cell level. Comparative analysis of a single- and a multicopy *gfp*-reporter for the colicin Ib promoter (P*cib*) revealed that the latter yielded optimal signal intensity for a diverse range of applications. We further validated this reporter and showed that *gfp* expression correlated well with colicin Ib (ColIb) protein levels in individual cells. P*cib* is negatively controlled by two repressors, LexA and Fur. Only a small fraction of *S*. Tm expressed *cib* under non-inducing conditions. We studied P*cib* activity in response to mitomycin C mediated DNA damage and iron limitation. Both conditions, if applied individually, lead to an increase in the fraction of GFP^+^ *S*. Tm, albeit an overall low fluorescence intensity. When both conditions were applied simultaneously, the majority of *S*. Tm turned GFP^+^ and displayed high fluorescence intensity. Thus, both repressors individually confine *cib* expression to a subset of the population. Taken together, we provide the first thorough characterization of a conventional *gfp*-reporter to study regulation of a group B colicin at the single cell level. This reporter will be useful to further investigate the costs and benefits of ColIb production in human pathogenic *S*. Tm and analyze *cib* expression under environmental conditions encountered in the mammalian gut.

## Introduction

Colicins are narrow-spectrum antimicrobials produced by members of the Enterobacteriaceae family (e. g. *E*. *coli*, *Salmonella spp*. and *Shigella*) and used for interference competition among close relatives. They serve as “public good” for the population of producers but are only synthesized by a small fraction of the population which lyse (and eventually die) and release colicins. This “division of labor” is supposed to increase the overall fitness of the producer population in competition against colicin-sensitive strains [1, 2]. Colicins bind specific outer-membrane receptors in order to kill susceptible target bacteria. Common killing mechanisms of colicins include pore formation (e.g. colicins A, B, E1, Ia, Ib, K, N and U), nuclease activity (e.g. colicins E2-E9) and the interference with peptidoglycan synthesis (colicin M) [3]. Colicins are classified in two groups (A and B) according to the mode of entry into target bacteria [3]. Group A colicins (A, E1 to E9, K, L, N, S4, U, and Y) translocate via the Tol system while group B colicins (B, D, H, Ia, Ib, M) use the TonB import pathway. Group A colicin-loci are encoded on small, high-copy plasmids and comprise a cluster of three genes: an activity gene encoding the colicin, an immunity gene to protect the producer population against self-killing and a lysis gene, required for cell lysis and concomitant colicin release.

We have recently shown that *Salmonella*-induced gut inflammation leads to parallel blooms of *Salmonella* and commensal Enterobacteriaceae in the mouse intestine [4]. *Salmonella enterica* serovar Typhimurium (*S*. Tm) strain SL1344 produces Colicin Ib (ColIb), a group B colicin. ColIb binds to the *E*. *coli* outer-membrane receptor CirA, translocates to the periplasm in a TonB-dependent fashion and kills by pore formation in the inner membrane [5–7]. The locus encoding the colicin activity gene (*cib*) and the gene for its corresponding immunity protein (*imm*) is located on the plasmid pColIB9 (86.9 kB; further termed p2) [8, 9]. In inflammation-inflicted blooms, ColIb confers a significant fitness benefit to *S*. Tm over competing commensal *E*. *coli* [9, 10].

*Cib* expression is tightly repressed to ensure, that the fraction of producers is kept at low rates under conditions, when colicin is not required. Most group A colicin promoters harbour two overlapping binding sites for the LexA repressor downstream of the −10 box. In the course of DNA damage and the consequent SOS-response, LexA is cleaved by the activated RecA protease and colicin expression is triggered. DNA damage was identified as main stimulus of colicin expression while other tested stressors alone (osmolarity, heat/cold shock, starvation) had a minor influence [11].

Rather, negative regulation by environmental signals seems to be an additional strategy for various colicins to ensure tight repression. This is termed “double-locking” [12]. *Cka* expression is growth phase dependent and induced by nutrient depletion and positively affected by ppGpp [13]. Further, the iron–sulphur cluster regulator IscR was shown to stabilize LexA at the promoter and is de-repressed upon nutrient starvation [12]. The lysis genes of ColE7 and ColE2 are controlled by CsrA in response to different carbon sources [14, 15]. Besides nutrient conditions, other environmental cues can also affect colicin expression. For example, comparison of ColE7 expression in biofilm and planktonic environments revealed a two to three-fold upregulation in biofilms [16]. Thus, signals derived from DNA damage, growth conditions and the nutritional status of the bacteria converge to regulate colicin expression.

**Fig 1:**
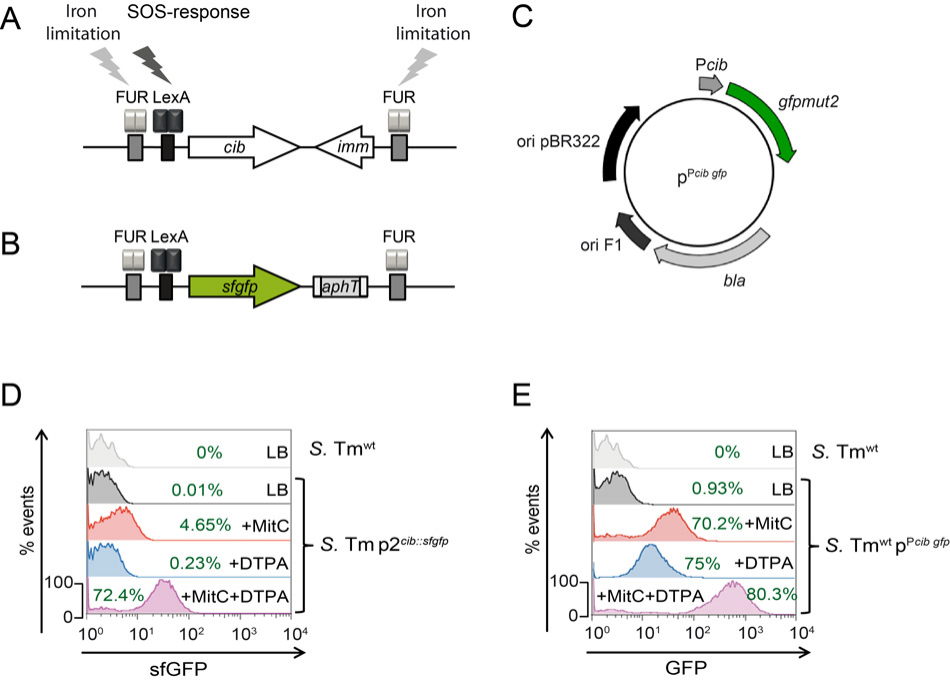
Characterization of the single- and multicopy *gfp*-reporter. **(A)** Genetic organization of the ColIb locus in *S*. Tm^wt^ and **(B)** the singlecopy reporter *S*. Tm p2^*cib*::*sfgfp*^. **(C)** Schematic view of the multicopy reporter p^P*cib gfp*^. **(D)** *S*. Tm p2^*cib*::*sfgfp*^ and **(E)** *S*. Tm p^P*cib gfp*^ were grown in LB (grey), or in LB supplemented with 0.25μg/ml MitC (red), 100μM DTPA (blue) or with both supplements (purple). Bacteria were analyzed for sfGFP or GFP -signal intensities by flow cytometry, respectively. *S*. Tm^wt^ lacking the reporter was used as negative control and for calculating the fraction (%) of GFP^+^ bacteria (green).

The colicin Ib promoter (P*cib*) harbors one LexA binding site [17] (Fig 1A). Furthermore, a putative binding site for Fur is located upstream of the -35 region which mediates iron-dependent repression of P*cib* {Nedialkova, 2014 #2}. Thus, Fur takes over a “double-locking” function for P*cib*. Accordingly, *cib* expression in *S*. Tm is maximally de-repressed under iron limitation and upon exposure to DNA damaging agents, such as the drug mitomycin C (MitC) and conditions prevailing in the inflamed intestine [10].

Quantitative analyses of regulation and expression dynamics at the single cell level can yield further insights into the evolutionary stabilization of lethal phenotypes such as production of colicins in bacterial populations. At the single cell level, expression has mainly been studied in case of group A colicins, including ColK, E7 and E2 [11, 17–19]. In this work, we studied ColIb production by *S*. Tm at the single cell level. We generated singlecopy and multicopy *gfp*-reporters for the colicin Ib promoter P*cib*. The multicopy reporter yielded optimal signal-intensity for a diverse range of applications and was therefore characterized and validated in detail.

## Materials and Methods

### Generation of bacterial mutant strains and plasmids

Bacterial strains and plasmids used in this study are listed in Table 1. *S*. Tm p2 ^*cib*::*sfgfp*^ (SJB15-2) and *S*. Tm p2^*cib-HA*^ (M1400) were generated by λ-Red recombination. To this end, plasmid pWRG7 or pSU315 [20] were used as templates for a *sfgfp* gene or HA (Hemagglutinin) -epitope tag flanked by a kanamycin-cassette (*sfgfp aphT* and HA *aphT*), respectively. The gene for superfolder GFP (*sfgfp*, [21]) was synthesized codon-optimized for expression in *Salmonella enterica* (Life Technologies, Regensburg, Germany). After amplification of the *sfgfp* gene using primers NotI-SFGFP-for and XhoI-SFGFP-rev (S1 Table), the resulting product was digested NotI/XhoI and subsequently cloned in the similarly-digested vector p2795 [22], yielding pWRG7. To construct *S*. Tm p2^*cib-HA*^, *sfgfp aphT* was amplified from pWRG7 by PCR using primers SFGFP_cib_fwd/SFGFP_cib_rev (S1 Table) and transformed into SB300 pKD46. Correct insertion was validated by PCR and by sequencing using primers Check up_SFGFP_fwd/ Check up_SFGFP /RFP _rev (S1 Table). The *cib*::*sfgfp aphT* allele was then transduced into a clean *S*. Tm^wt^ (SB300 pWKS30) background by P22-transduction [23] to generate *S*. Tm p2^*cib*::*sfgfp*^. To construct *S*. Tm p2^*cib-HA*^ (M1400), the HA *aphT* epitope was amplified by PCR from pSU315 using primers Colicin-HA-fwd/Colicin-HA-rev (S1 Table). Correct insertion was validated by PCR and by sequencing using primers Col-ÜE-XbaI/ Col-ÜE-XhoI (S1 Table). The *cib-HA aphT* allele was then transduced into a fresh *S*. Tm^wt^ (SB300 pWKS30) background by P22-transduction [23]. Expression of the HA-tagged ColIb in *S*. Tm^wt^ p2^cib-HA^ was verified by Western Blot using HA-specific antiserum (Santa Cruz). ColIb-HA shows similar bactericidal activity against sensitive strains as untagged ColIb and can therefore be considered as functional (not shown). To construct the plasmid pSJB16 (p^P*cib*^), pM1437 (p^P*cib gfp*^) was hydrolyzed by EcoRI to remove the *gfpmut2* gene and then re-ligated. A similar approach was employed to construct the control plasmid pSJB17 (p^control^): pM968 (p^*gfp*^) was hydrolyzed by EcoRI, relegated and the *gfpmut2* gene removed.

**Table 1:** Bacteria and plasmids used in this study.

*S*. Tm *strains*	*Designation*	*Description/genotype*	*Reference*
SB300	*S*. Tm^wt^	*S*. Tm SL1344, Sm^R^	[24]
M1400	*S*. Tm p2^*cib-HA*^	*S*.* *Tm SL1344 *cib*-*HA-aphT*, Sm^R^, Kan^R^	This study
SJB15-2	*S*. Tm p2^*cib*::*sfgfp*^	*S*.* *Tm SL1344 *cib imm*::*sfgfp-aphT* Sm^R^, Kan^R^	This study
***E*. *coli strains***			
DH5α	*Ec* ^DH5α^	F^-^ Φ80*lac*ZΔM15 Δ(*lac*ZYA-*arg*F) U169 *rec*A1 *end*A1 *hsd*R17(r_k_^-^, m_k_^+^) *pho*A *sup*E44 *thi*-1 *gyr*A96 *rel*A1 λ^-^	Invitrogen
MG1655	*Ec* ^*MG1655*^	*E*.* coli* K-12 strain MG1655, F- lambda- *ilvG*- *rfb*-50 *rph*-1; Rif^R^, Sm^R^	[25, 26]
***Plasmids***			
pWKS30		Low-copy vector; pSC101-based replicon; ampicillin-resistance marker	[27]
pKD46		Temperature sensitive replication (repA101ts); encodes lambda Red genes (*exo*, *bet*, *gam*); native terminator (tL3) after *exo* gene; arabinose-inducible promoter for expression (P_araB_); encodes *araC* for repression of P_araB_ promoter; ampicillin resistance	[28]
pWRG7		High-copy vector, colE1-replicon; carries *sfgfp aphT* flanked by FRT sequences; ampicillin resistance	This study
pSU315		Low-copy vector; R6K gamma replicon	[20]
pSJB17	p^control^	pBR322-derivative, ampicillin resistance	This study
pSJB16	p^Pcib^	pBR322-derivative, P*cib*, ampicillin resistance	This study
pM968	p^*gfp*^	pBR322-derivative, promoterless, *gfpmut2;* ampicillin resistance	[29]
pM1437	p^Pcib gfp^	pBR322-derivative, P*cib*, *gfpmut2*; ampicillin resistance	[10]
pM979	p^Prpsm gfp^	pBR322-derivative, P*rpsM*, (ribosomal *rpsM* promoter, constitutive), *gfpmut2*, ampicillin resistance	[29]
pM974	p^PsicA gfp^	pBR322-derivative, P*sicA* (locus in *Salmonella* pathogenicity island 1), *gfpmut2*, ampicillin resistance	[29, 30]

### Bacterial growth conditions

If not otherwise stated, *S*. Tm and *E*. *coli* strains were grown in 3ml LB medium for 12h under mild aeration at 37°C in test tubes in a rotor wheel. Antibiotic concentrations used were ampicillin (100μg/ml), streptomycin (50μg/ml) and kanamycin (30μg/ml). The (o.n.) culture was then used for inoculation (1:20) of 3ml LB subcultures. The subcultures were supplemented with antibiotics and with either 0.25μg/ml mitomycin C (MitC; Roth) or with 100μM of diethylene triamine pentaacetic acid (DTPA; Sigma) or both as described [10], and grown for 4h at 37°C under mild aeration.

### Determination of plasmid stability

The *S*. Tm^wt^ p^Pcib gfp^ strain was inoculated on LB (Sm^50^, Kan^30^) from -80°C cryostock and incubated o.n. at 37°C. The following day, a single colony was used to inoculate 10ml LB liquid medium (no antibiotics). This culture was incubated o.n. at 37°C with 180rpm shaking. Subsequently, a sample of 1ml for an OD_600_ of 0.4 was taken and used to set up a subculture in 10ml LB liquid media (no antibiotics). This subculture was incubated until OD_600_ = 2–3. Four more passages were carried out in a similar fashion. The experiment was done in triplicates.

From each passage (1–5), samples were taken (OD_600_ of 1), diluted and plated on LB agar plates to obtain single colonies. A minimum of 100 colonies were screened for ampicillin resistance as a measure for p^Pcib gfp^ plasmid stability.

### Determination of plasmid copy number

To determine plasmid copy number of p^Pcib gfp^_,_ total DNA was extracted from bacterial cultures of *S*. Tm^wt^ p^Pcib gfp^ strain (passage 1–5). Bacteria were harvested by centrifugation and resuspended in 567μl TE-buffer (10 mM Tris-Cl, 1 mM EDTA, pH 8.0), 30μl 10% SDS and 3μl proteinase K (20 mg/ml) and incubated for 1h at 55°C. Thereafter, 100μl of 5M NaCl and 80μl CTAB/NaCl (4.1g NaCl and 10g CTAB [Cetyltrimethylammoniumbromid] dissolved in 100ml _dd_H_2_O) were added and incubated at 65°C for 10min. Subsequently, DNA was extracted with an equal volume of phenol/chloroform/isoamylalcohol (25:24:1) and EtOH precipitated. DNA was suspended in 60μl TE-buffer and the DNA concentration was determined using a NanoDrop Spectrophotometer ND-1000 (PEQLAB Biotechnology) and used for qPCR.

qPCR was performed using FastStart Essential DNA Green Master reaction mix for SYBR Green I-based real-time PCR (Roche) on a LightCycler^®^ 96 Instrument. Each reaction was done in triplicates in a reaction mix of 20μl containing: 10μl of 2 x FastStart Essential DNA Green Master (Roche), 0.2μl forward primer (0.3μM), 0.2μl reverse primer (0.3μM), 2.5μl of total DNA (5-10ng/ μl) and 7.1μl RNase-free water. The following oligonucleotides were used: Ampli1_Fwd/Ampli1_Rev for p^Pcib gfp^ (amplicon: 73bps) and PsicA_2_fwd/PsicA_2_rev, specific for the *sicA* promoter which is present at one copy /*S*. Tm genome (amplicon: 79bps). The reaction was started with 1 cycle 95°C for 10min, following a 3-step amplification for 45 cycles at 95°C for 10s, 50°C (p^Pcib gfp^) or 54°C (16S rRNA) and elongation at 72°C for 10s. To determine the linear dynamic range and reaction efficiency, a standard curve was generated using a 1:10 dilution series of the linearized plasmid p^Pcib gfp^ or linearized plasmid pM974 (harboring the p*sicA*) from 2.5 x 10^7^ to 1.0 x 10^2^ copies for p^Pcib gfp^, (efficiency 98%) and 2.5 x 10^7^ to 1.0 x 10^−1^ copies in case of pM974 (efficiency 93%). Plasmids were linearized, purified and DNA was quantified using Quant-IT Picogreen dsDNA assay kit (Life Technologies). The standard curves were used for absolute quantification of genome and plasmid copy numbers. From this data, the relative copy number of p^Pcib gfp^ per *S*. Tm genome was calculated.

### Generation of samples for immunoblotting

Total bacterial protein was harvested from subcultures (250μl of an OD_600_ = 1). Cells were collected by centrifugation (4°C, 10min, 10.621xg) and pellets were suspended in 250μl 1x loading buffer (50mM Tris-HCl pH 6.8, 100mM DTT, 2% SDS, 0.1% bromphenole blue, 10% glycerol) and lysed by incubation at 95°C for 10min. For supernatant samples, 500μl of subculture were pelleted and 400μl culture supernatant were added to 5x loading buffer and boiled for 10min at 95°C.

### SDS-polyacrylamide gel electrophoresis (PAGE) and immunoblotting

SDS-PAGE was used to separate proteins [31]. Subsequently, proteins were transferred using a semidry blot onto a nitrocellulose membrane (GE Healthcare). The membrane was blocked (1x PBS, 0.1% Tween, 5% milk powder) and developed with polyclonal rabbit-anti HA (Y11, Santa Cruz) antiserum and goat-anti-rabbit-HRP (GE Healthcare) as secondary antibody followed by detection with the ECL detection system (GE Healthcare).

### Colicin killing-assay

Colicin production and sensitivity was assayed as described [10]. Briefly, the colicin producing strain was grown o.n. as small spot (Ø 5mm) on LB agar containing 0.25μg/ml MitC (Roth). The plate was overlaid with the tester strain (Ec^MG1655^) in top-agar (0.75% agar). Growth of tester strain was analyzed after 24h. Formation of an inhibition zone (halo) around the producer indicated production of colicin and sensitivity of the tester strain.

### Immunofluorescence staining of intrabacterial proteins and confocal microscopy

Immunofluorescence staining of intrabacterial proteins was performed as described [32]. Bacteria were grown as described above in sterile polystyrene test tubes (Cultube, Simport). 250μl (OD_600_ 1) culture were spun down for 5min, 4°C, 8000 rpm. Pellets were resuspended with 250μl ice cold 1x phosphate buffer saline (PBS) and 750μl of ice cold 4% paraformaldehyde (PFA) in PBS and incubated for 1h on ice. Subsequently, bacteria were washed three times with ice-cold PBS. Fixed bacteria were immobilized on poly L-lysine coated glass slides (Superfrost Plus, Thermo Scientific) by drying. Immobilized bacteria were additionally fixed for 5min with 4% PFA in PBS and washed 3 times with PBS. Bacteria were treated for 5min with permeabilization buffer A (20mM Tris, 0.1% TritonX-100, 50mM EDTA, 1.8% Glucose; pH 8.0), washed 3 times in ice cold permeabilization buffer B (25mM Tris, 10mM EDTA and 1.8% Glucose; pH 8.0), and incubated for 30min with permeabilization buffer B supplemented with 5mg/ml lysozyme at 4°C. Afterwards, bacteria were washed and blocked with blocking solution (10% normal goat serum diluted in PBS) for 1h. Bacteria were stained with primary antibody (rabbit-anti HA Y11 [1:200] (Santa Cruz); mouse-anti DnaK [1:200] (Enzo Life Sciences) or rabbit-anti GFP [1:200] (antibodies-online.com) diluted in blocking solution and washed 3 times with PBS. Thereafter, bacteria were stained with secondary antibodies (anti-rabbit-Dylight549-conjugate [1:400] (Jackson) or anti-mouse-RhodamineRed-X-conjugate [1:200] (Invitrogen) diluted in blocking solution. Bacterial DNA was stained with 4’6-Diamidine-2-phenylindol (DAPI [1μg/ml], Roth) or Hoechst 33342 ([10μg/ml], Thermo Scientific). Subsequently, bacteria were washed, dried in the dark and mounted with Vectashield (Vector) and sealed with nail varnish.

### Confocal microscopy and image analysis

Using 63x oil objective and a magnification of 1 or 2.4 a minimum of 3 images were taken with a Leica SP5 confocal microscope. Image analysis was done using the ImageJ software, version 1.48v (Wayne Rasband, National Institute of Health, USA) [33]. Bacteria were detected in the DAPI channel. A mask was created to define objects (bacteria). This mask was superimposed on the green (GFP-signal) and red (ColIb-HA- or anti-GFP-signal) channels. The cell size, the integrated density and the mean fluorescence intensity (MFI) were calculated for each object. To correlate intrinsic GFP-fluorescence with signals of fluorescently labeled ColIb-HA or GFP, respectively, the corrected total cell fluorescence (CTCF) of the objects was calculated according to the formula: Integrated Density of the selected object–(Area of the selected object X Mean fluorescence of background signal). The detection limit for both MFI and CTCF values is given as the maximum value of MFI or CTCF signal determined for a control strain.

### Flow Cytometry

Bacteria were grown as described and diluted in filtered PBS to a concentration of 10^7^cfu/ml. Data were recorded by a FACS Canto II running the FACSDiva software (Aria Becton Dickinson). Data was analyzed using the FlowJo software (Tree Star, Inc.).

### Statistical analysis

Statistical analyses were performed with Graph Pad Prism Version 5.01. To calculate statistical significance, the Kruskal-Wallis test with Dunn’s Multiple Comparison test was performed. *P*-values less than 0.05 were considered as significant. For correlation the Spearman-rank coefficient [ρ] was calculated.

## Results and Discussion

### Comparison of single-and multicopy *gfp*-reporters for ColIb gene expression at the single cell level

In order to analyze the expression of the ColIb gene (*cib*) at the single cell level, we generated single and multicopy P*cib gfp*-reporters. For the singlecopy reporter (*S*. Tm p2^*cib*::*sfgfp*^) the entire ColIb locus on p2 (*cib* and *imm*) was exchanged against superfolder *gfp* (*sfgfp*) [21], which encodes a bright and photostable GFP-variant (Fig 1B). The singlecopy reporter strain was characterized by flow cytometry under different environmental conditions. *S*. Tm p2^*cib*::*sfgfp*^ was grown for 4h to late logarithmic growth phase either in LB without supplements or in LB supplemented with 0.25μg/ml MitC (to induce DNA damage) or 100μM DTPA (to induce Fe^2+^-limitation) or both supplements. Flow cytometric analysis showed that the single copy reporter is induced under iron limiting and SOS-inducing conditions and that *cib* expression was maximal if both supplements were added (Fig 1D). In conclusion, signal intensity of the singlecopy reporter was rather low, which is in line with the notion, that pColIB9 is only present in one copy per cell.

As fluorescence-intensity of the singlecopy reporter was overall low, we also generated a multicopy reporter by fusing the promoter region P*cib* to *gfp* in a derivative of the pBAD24 *ori* pBR322 [34] (Fig 1C). pBR322 is a derivative the ColE1-type plasmid pMB1 [35] and shares the replication control mechanism with ColE1 and relatives. Intriguingly, p^Pcib gfp^ is therefore the descendant of a group A colicin plasmid.

As expected, the mean signal intensity was higher compared to the single copy reporter under all conditions (Fig 1E). Consequently, the fraction of GFP^+^ *S*. Tm was consistently higher for the multicopy reporter, while the overall pattern of the GFP^+^ *S*. Tm population under different conditions strongly resembled the singlecopy reporter. The data is in accordance with previous results obtained by bulk assays (Western blot and luciferase reporter) [10].

Intriguingly, different studies on colicin-expression rates in bacterial populations reported rather similar results although media composition, growth conditions and the detection limit of the fluorescent protein (FP) likely varies between different laboratories and analysis methods. Using *gfp*-reporters based on a low copy number pSC101 plasmid, a rate of ~0.5% was determined for ColA, ColN and ColE1 in the early stationary phase in rich media [36]. For ColE7, the same study determined 1.5% GFP^+^ cells in the early stationary phase. In a different study using the same *gfp*-reporter, comparable levels were determined in the stationary phase and slight increases were observed at post-exponential (1.8±0.2%) and exponential phase (2.3±1.2) [16]. For ColK, 3% of the *E*. *coli* population was GFP^+^ in stationary cultures using a reporter based on the natural colicin K-encoding plasmid [19].

Our FACS results using the multicopy *gfp*-reporter p^Pcib gfp^ showed that 0,93% of the *Salmonella* population were GFP^+^ in LB during late exponential phase. Measurements using the singlecopy reporter yielded much lower rates (0,01%). This demonstrates that plasmid copy number can introduce a major bias to quantification of *gfp* expression rates in bacterial populations.

### Effect of inducer titration on *cib* expression in *S*. Tm

As previously shown for other colicins [19, 36], a small fraction of the *S*. Tm population carrying the reporters was GFP^+^ in the absence of supplements (DTPA, MitC). Colicin induction in this subpopulation could be due to spontaneous induction of the SOS-response [37]. Similarly, the noise observed in prophage activation in *Corynebacterium glutamicum* was also attributed to a spontaneous SOS-response in about 0.2% of cells [38].

When either MitC or DTPA (or both inducers) were added, the entire *S*. Tm population responded with *gfp* expression (heterogeneous expression pattern) as shown with both reporters (Fig 1D and 1E). In fact, similar observations were made for ColK. Under MitC or when LexA was defective nearly all cells of the population expressed the *cka-gfp* fusion and turned GFP^+^ [19]. Similar observations were made when the LexA binding site of the *cka* promoter was modified [37]. On the basis of the “division of labor” hypothesis for colicin production, we expected to observe a bimodal expression pattern for a colicin under inducing conditions. In our experiments we used rather non-physiological supplement concentrations optimized for bulk assays to maximally induce P*cib*. To test, if lower supplement concentrations would trigger a bimodal expression pattern (i.e. two distinct populations), we generated dose-response curves for the two different supplements MitC and DTPA using the singlecopy reporter *S*. Tm p2^*cib*::*sfgfp*^ (S1 Fig). Curiously, bimodal expression was never observed, not even at low supplement concentrations. We reasoned that due to the presence of two different repressor-binding sites (FUR- and LexA-box) in P*cib*, bimodal expression might require both inducing agents at the same time. Therefore, one supplement was kept at a constant concentration (ranging from low to high) while the other supplement was titrated. However, under all possible combinations, fluorescence intensities were log-normally distributed and bimodality (e.g. two peaks of different fluorescence intensity) was not observed (S1 Fig). The experiments were repeated using *S*. Tm p^Pcib gfp^ (multicopy *gfp*-reporter strain) and similar results were obtained (S1 Fig). In conclusion, *cib* expression in *S*. Tm under the tested *in vitro* conditions does not appear to be bimodal. As the mechanism of ColIb release is still unknown, the actual costs of ColIb production cannot be determined (e.g. release by lysis or secretion). Thus, it is unclear to which extend the principle of “division of labor” applies for *cib* expression. Further work will be needed to clarify this issue.

### The multicopy *gfp*-reporter is stable and does not influence intrinsic *cib* expression in *S*. Tm

The major advantage of the multicopy-*gfp*-reporter is its high sensitivity. However, the reporter plasmid might be lost due to plasmid instability and thereby bias results. To address this, we determined stability of p^Pcib gfp^ in *S*. Tm^wt^ in the absence of antibiotics. Plasmid stability was >99% after 24 generations during 5 successive passages in LB without ampicillin. Similarly, the copy numbers of p^Pcib gfp^ in *S*. Tm^wt^ was stable at the different rounds of passaging (S2 Fig). From this we concluded, that p^Pcib gfp^ stability is sufficient for the planned applications.

**Fig 2:**
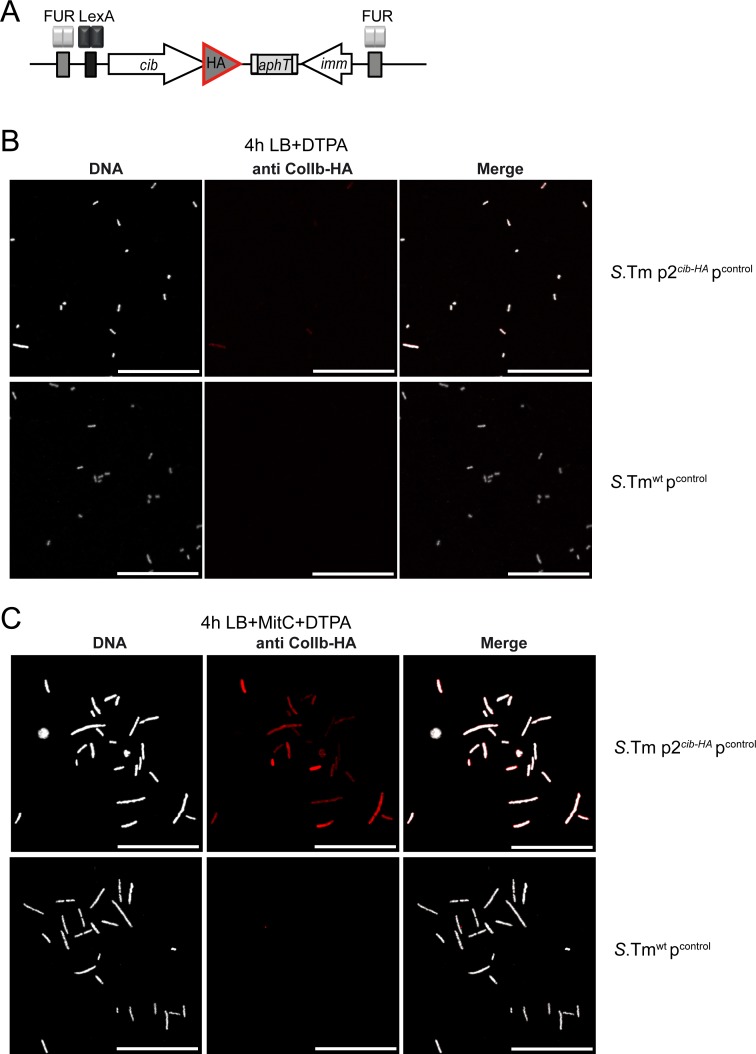
Detection of ColIb-HA within individual bacteria. **(A)** Schematic of the modified ColIb locus in the strain *S*. Tm p2^*cib-HA*^. *S*. Tm p2^*cib-HA*^ p^control^ and *S*. Tm^wt^ p^control^ were grown in LB supplemented with 100μM DTPA **(B)** or LB supplemented 0.25μg/ml MitC + 100μM DTPA **(C)**. Bacteria were fixed, permeabilized and intracellular ColIb-HA was detected using HA-specific antiserum (red). DNA was stained with DAPI (greyscale). Scale bar 25μm.

*S*. Tm^wt^ carrying the multicopy reporter retains its native, functional ColIb locus in addition to the reporter. To test if *gfp* expression of the multicopy reporter (p^Pcib gfp^) correlates with intrinsic *cib* expression, we generated a chromosomal ColIb-HA fusion construct in *S*. Tm ^wt^ which could be detected by immunofluorescence (*S*. Tm p2^*cib-HA*^; Fig 2A). ColIb*-*HA still retains full bactericidal activity against a ColIb-sensitive *E*. *coli* strain as determined by halo-assay (not shown). ColIb*-*HA could be detected within lysozyme-permeabilized bacteria by intrabacterial immunofluorescent staining (Fig 2B and 2C). Efficiency of lysozyme permeabilization was optimized and antibody-specificity confirmed for this assay (S3 Fig; Fig 2B and 2C).

By introducing >100 copies of the promoter P*cib* or the *gfp* gene (S2 Fig), the multicopy reporter may affect regulation of the native P*cib*. Therefore, we aimed to address if *cib* expression is influenced by multicopy effects of p^Pcib gfp^. We generated several derivatives of p^Pcib gfp^ (S4 Fig). The plasmid p^control^ lacking the P*cib* element and *gfp* and only retaining the vector backbone served as negative control (S4 Fig). Furthermore, two additional control plasmids were constructed: p^Pcib^ lacking the *gfp* gene (S4 Fig) and p^*gfp*^ lacking the *cib* promoter P*cib* (S4 Fig). Expression of fluorescent proteins can negatively influence bacterial fitness [39, 40]. To address any possible effects of *gfp* expression on native P*cib*, we also employed a plasmid for constitutive high-level *gfp* expression p^Prpsm gfp^ (S4 Fig).

**Fig 3:**
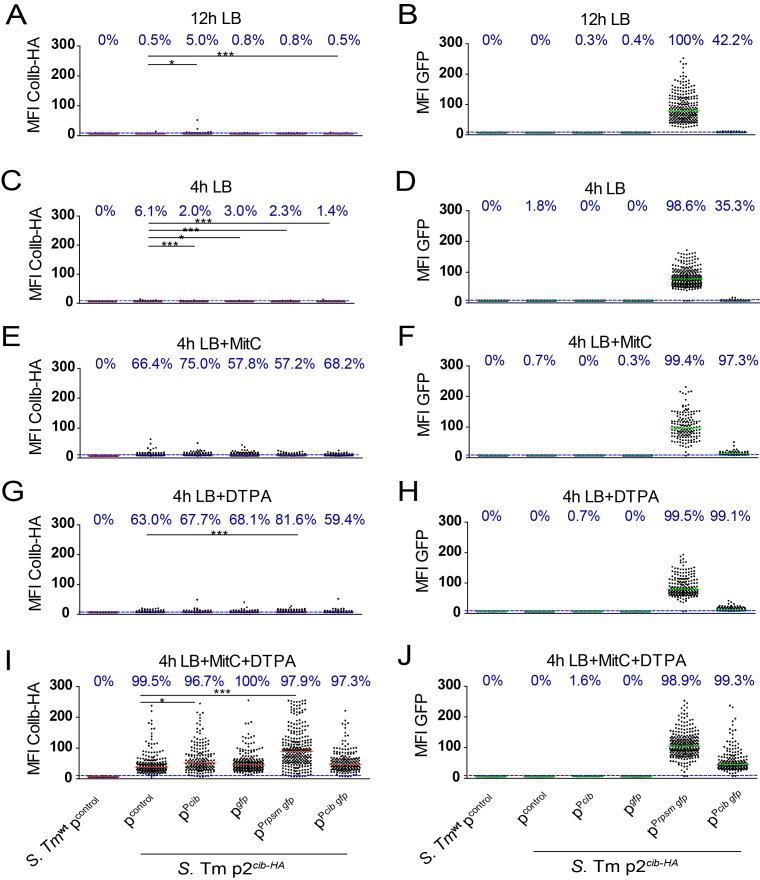
Influence of the multicopy reporter p^Pcib gfp^ and its derivatives on expression of native *S*. Tm p2^cib-HA^ at the single cell level. *S*. Tm p2 ^*cib-HA*^ transformed with p^Pcib gfp^ or the derivatives (p^control^, p^Pcib^, p^*gfp*^ and p^Prpsm gfp^), respectively, were grown in LB (+ampicillin) overnight (12h; stationary phase) or subcultured for 4h in LB (late logarithmic phase) with or without indicated supplements (0.25μg/ml MitC, 100μM DTPA). Bacteria were fixed, permeabilized and intracellular ColIb-HA was detected using HA-specific antiserum and a DyLight 594-conjugated secondary antibody, DNA was stained with DAPI. Bacteria were imaged by confocal microscopy and fluorescence of DAPI, GFP and DyLight 594-conjugate was recorded. Mean fluorescence intensity (MFI) of ColIb-HA-DyLight594 **(A,C,E,G,I)** and GFP **(B,D,F,H,J)** as determined by ImageJ is shown. Dots represent MFI of individual objects (bacteria). Bars represent the median and the dotted line the detection limit (background fluorescence of *S*. Tm^wt^ p^control^). Values indicate the fraction (%) of the population above detection limit (blue). Statistical analysis was done using Kruskal-Wallis test with Dunn's post test (* *p* < 0.05).

*S*. Tm p2^*cib-HA*^ was then transformed with p^Pcib gfp^ or one the four derivatives, respectively. To quantify *cib-*HA expression under different environmental conditions, bacteria were grown in LB with or without addition of supplements to late logarithmic growth phase. Additionally, bacteria from a stationary culture (12h grown in LB) were included. Bacterial samples were fixed, permeabilized and DNA and ColIb-HA were stained and analyzed by immunofluorescence microscopy (Fig 2B and 2C). Fluorescence intensity of intrabacterial ColIb-HA levels (Dylight 549 fluorescence) and GFP were quantified using ImageJ (Fig 3).

In LB without supplements, ColIb-HA and GFP levels were close to the detection limit of this method (Fig 3A–3D). Overall, the relative rates of GFP^+^ *S*. Tm were different when compared to the FACSs analysis (Fig 1). This is likely due to different thresholds applied for detection of GFP^+^ *S*. Tm cells. Under any of the *cib*-inducing conditions, no major differences in ColIb-HA levels between *S*. Tm p2 ^*cib-HA*^ carrying the multicopy reporter p^Pcib gfp^ and the derivatives p^control^, p^*gfp*^ and p^Pcib^ were detected (Fig 3E–3J). Thus, we concluded that the multicopy reporter (p^Pcib gfp^) does not evidently influence expression of native *cib* under the tested conditions. In contrast, we found that carriage of p^Prpsm gfp^ is accompanied by increased ColIb-HA levels (Fig 3I). This suggests that GFP, or overexpressed proteins in general, may trigger bacterial stress-responses at very high protein concentrations. Alternatively, the requirement for specific tRNAs that become limiting due to the GFP synthesis may account for increased stress.

### Correlation of *gfp* expression of the reporter p^Pcib gfp^ with intrinsic *cib-*HA expression

**Fig 4:**
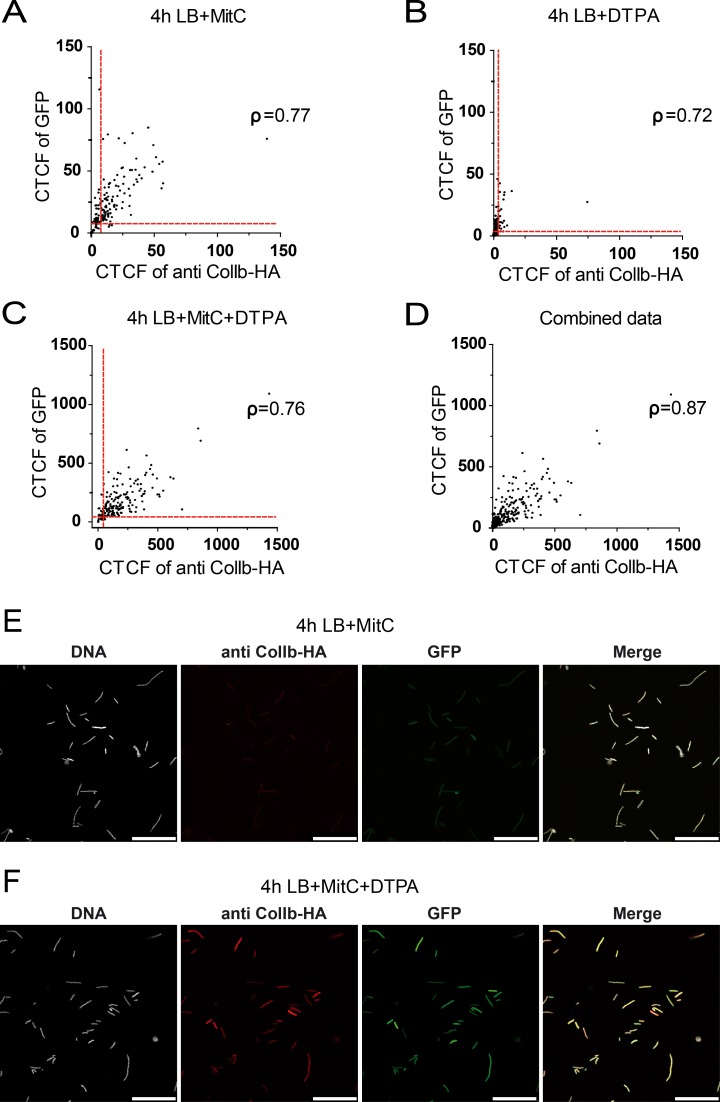
Correlation of *gfp* expression of the reporter p^Pcib gfp^ with intrinsic *cib*-HA expression under different conditions at the single cell level. *S*. Tm p2 ^*cib-HA*^ transformed with p^Pcib gfp^ was grown in the presence of different supplements as described in Fig 4. Bacteria were fixed, permeabilized and intracellular ColIb-HA was detected using HA-specific antiserum, DNA was stained with DAPI. Bacteria were imaged by confocal microscopy and fluorescence of DAPI, GFP and the DyLight 594-conjugate was recorded. Corrected total cell fluorescence (CTCF) was calculated and GFP-fluorescence of individual bacteria was correlated to Dylight549 fluorescence **(A-C).** A combination of data from all 3 conditions is shown in **(D)**. Red line: detection limit; [ρ] = Spearman-rank correlation coefficient. Examples are shown for intrabacterial IF for *S*. Tm supplemented with MitC **(E)** and MitC+DTPA **(F)**. Scale bar 25μm.

Next, we set out to determine if *gfp* expression of the multicopy reporter p^Pcib gfp^ is a valid proxy for inferring native *cib*-HA expression in individual bacteria. Formation of the GFP-chromophore depends on the correct folding of the protein. Thus, slow maturation of newly synthesized unfolded GFP-molecules could negatively affect correlation. To address this, we tested if GFP-fluorescence (GFP^fl^; mature folded GFP) correlated with GFP-protein (total GFP) using a GFP-specific antiserum and intrabacterial immunofluorescence microscopy. This experiment revealed that GFP^fl^ correlates well with GFP protein levels in *S*. Tm under all conditions tested, indicating fast protein maturation (overall Spearman-rank correlation [ρ] = 0.95; S5 Fig). Next, we correlated *gfp* expression of the reporter p^Pcib gfp^ with *cib*-HA expression in individual bacteria (Fig 4). To this end, we plotted the GFP-signal against ColIb-HA-DyLight594 signal of individual *S*. Tm p2^*cib-HA*^ p^Pcib gfp^ from the experiment depicted in Fig 4. GFP-levels correlated well to *cib-*HA expression under *cib*-inducing conditions (overall Spearman-rank correlation [ρ] = 0.87; Fig 4D).

## Conclusion

In summary, we provide a comprehensive characterization of reporter tools to study regulation of the group B colicin ColIb at the single cell level in the human enteric pathogen *S*. Tm. Taken together, the multicopy reporter p^Pcib gfp^ yields a high GFP signal and closely reflects expression of native *cib*. We could rule out that the multicopy-reporter induces substantial changes in regulation of the native ColIb locus at the single cell level, which might have been caused by the introduction of additional copies of the P^*cib*^ promoter (including the repressor binding sites). Additionally, toxic effects of GFP might cause bacterial stress responses leading to an erroneous upregulation of the reporter gene [39, 40]. However, we show that intrinsic ColIb-HA levels correlate with the FP-reporter which establishes p^Pcib gfp^ as a powerful tool to analyse *cib* expression at the single cell level. Therefore, this reporter can further be used for time lapse microscopy experiments in microfluidics platforms as well as for studying *cib* expression *in vivo* in the murine gut.

## Supporting Information

S1 Fig Influence of the supplement concentration on *cib* expression of *S*. Tm p2^*cib*::*sfgfp*^ and *S*. Tm p^Pcib^. *S*. Tm p2^*cib*::*sfgfp*^ (single copy *gfp*-reporter) was cultured for 4h in LB with either increasing concentrations of **(A)** DTPA (6μM, 12μM, 25μM, 50μM, 100μM and 200μM), while MitC concentration was kept constant at 0μg/ml, 0.01μg/ml, 0.1μg/ml or 0.25μg/ml or **(B)** MitC (0.01μg/ml, 0.05μg/ml, 0.1μg/ml 0.2μg/ml, 0.25μg/ml, 0.5μg/ml and 1μg/ml) while the DTPA concentration was kept constant at 0μM, 6μM, 25μM or 100μM. Bacteria were subsequently analyzed by FACS for GFP-signal intensity. Cultures of *S*. Tm p^Pcib^ (multi-copy *gfp*-reporter) were grown in LB with either increasing concentrations of **(C)** DTPA (6μM, 12μM, 25μM, 50μM, 100μM and 200μM), while MitC concentration was kept constant at 0.25μg/ml or **(D)** MitC (0.01μg/ml, 0.05μg/ml, 0.1μg/ml 0.2μg/ml, 0.25μg/ml, 0.5μg/ml and 1μg/ml) while the DTPA concentration was kept constant at 100μM. Bacteria were analyzed by FACS for GFP-signal intensity. S. Tm^wt^ lacking the reporter was used as negative control and for calculating the fraction (%) of GFP+ bacteria (green).
(TIF)

S2 Fig Copy number and plasmid stability of the multicopy *gfp* reporter p^Pcib gfp^. *S*. Tm^wt^ harboring p^Pcib gfp^ was cultured for five consecutive passages (1–5) in 10ml LB. Briefly, a sample of 1ml for an OD_600_ of 0.4 was taken and used to set up a subculture in 10ml LB liquid media (no antibiotics). This subculture was incubated until OD_600_ = 2–3. Four more passages were carried out in a similar fashion. From each passage (1–5), samples were taken (1ml from 1 OD_600_) and total DNA was extracted for quantitative PCR analysis. **(A)** Copy number of p^Pcib gfp^ per ml culture (OD_600_ of 1) as determined by absolute quantification. **(B)** Genome copy number as determined by absolute quantification of P*sicA* copies per ml culture (OD_600_ of 1). Data were analyzed by 1-way ANOVA. No significant differences were determined between passages. p^Pcib gfp^ copy number in *S*. Tm^wt^ in the 5 consecutive passages (mean±SD) as calculated from data shown in A and B: passage 1: 169±91; passage 2: 84±18; passage 3: 147±57; passage 4: 198±100; passage 5: 127±67.
(TIF)

S3 Fig Validation of intrabacterial immunostaining for DnaK and ColIb-HA. *S*. Tm p2^*cib-HA*^ was grown in LB for 12h and 4h in LB or for 4h supplemented with MitC, DTPA or both. The efficiency of lysozyme permeabilization was validated by staining the cytosolic, constitutively expressed protein DnaK in lysozyme-treated (lower panel) and non-treated samples (upper panel) **(A)**. ColIb-HA was detected within a small fraction of *S*. Tm grown in LB after lysozyme treatment (arrow) but not in untreated samples (upper panel) **(B)**. ColIb-HA was detected in *S*. Tm grown in LB MitC+DTPA after lysozyme treatment (lower panel) and also in a small fraction of cells without lysozyme treatment (upper panel) **(C)**. Scale bar 25μm.
(TIF)

S4 Fig Plasmid maps of the multicopy reporter p^Pcib gfp^ and its derivatives. Plasmid maps of **(A)** p^Pcib gfp^ (multicopy reporter); **(B)** p^control^ (only plasmid backbone) **(C)** p^Pcib^ (only promoter of *cib*); **(D)** p^*gfp*^ (only *gfp*); **(E)** p^Prpsm gfp^ (constitutive promoter) are shown.
(TIF)

S5 Fig Correlation of GFP-fluorescence with anti-GFP immunofluorescent staining. *S*. Tm^wt^ p^Pcib gfp^ was grown for 12h **(A)** and 4h **(B)** in LB or for 4h supplemented with MitC **(C)**, DTPA **(D)** or both **(E)**. Bacteria were fixed, lysozyme permeabilized and stained with a GFP-specific antiserum and a Dylight549-conjugated secondary antibody and analyzed by fluorescence microscopy followed by image analysis. Corrected total cell fluorescence (CTCF) was calculated and GFP-fluorescence of individual bacteria was correlated to Dylight549 fluorescence ([ρ] Spearman-rank correlation coefficient). A combination of all data is shown in **(F)**. Red line: detection limit. Examples for IF-microscopy are shown in **(G)** and **(H)**. Scale bar 25μm.
(TIF)

S1 Table Primers used in this study. (DOCX)
