# Supplementary figures and images for: Reporters for Single-Cell Analysis of Colicin Ib Expression in Salmonella enterica Serovar Typhimurium
Source: PLoS One. 2015 Dec 10;10(12):e0144647. doi: 10.1371/journal.pone.0144647 (PMC4675545; doi:10.1371/journal.pone.0144647)

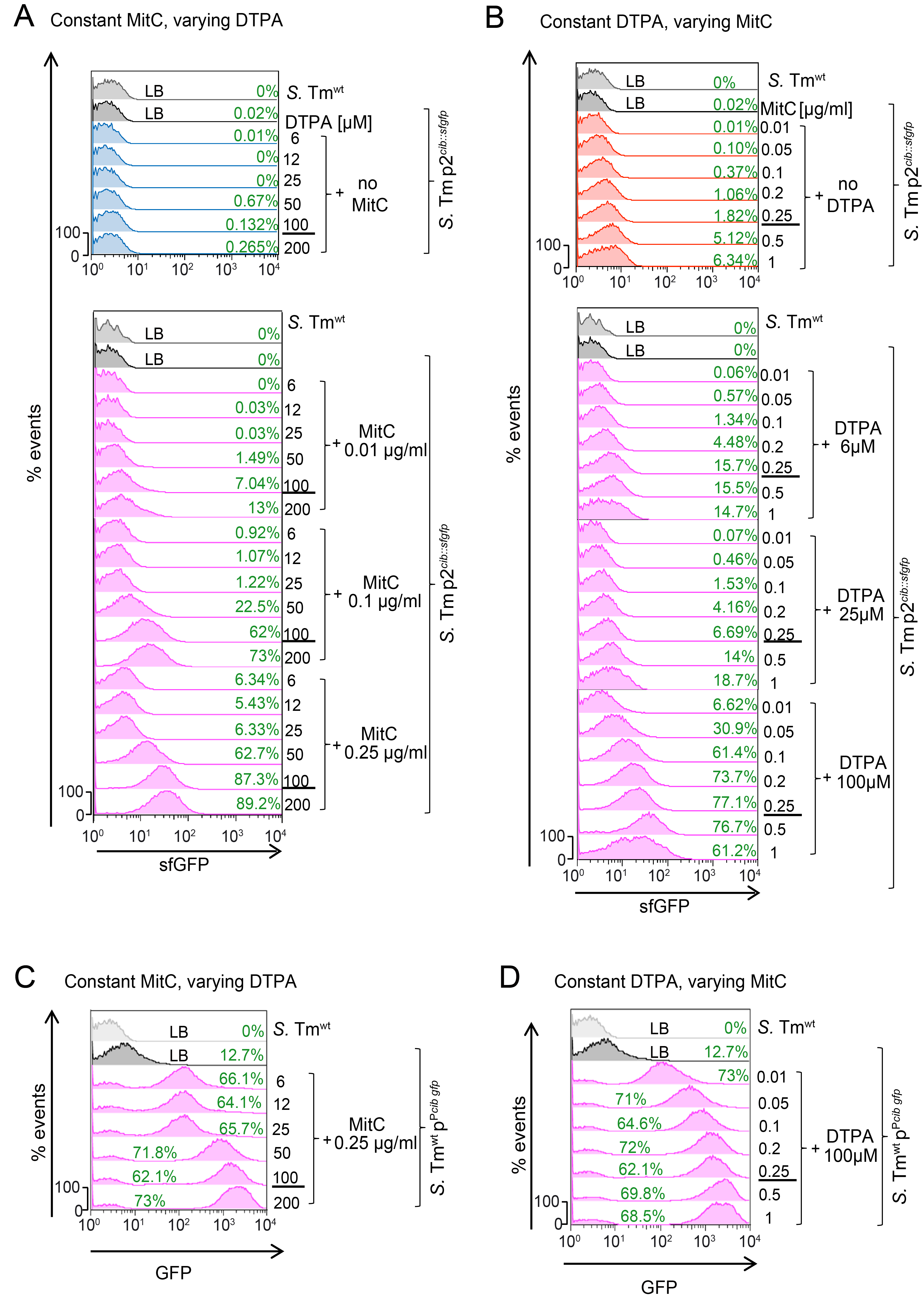

Supplement: S1 Fig — S. Tm p2cib::sfgfp (single copy gfp-reporter) was cultured for 4h in LB with either increasing concentrations of (A) DTPA (6μM, 12μM, 25μM, 50μM, 100μM and 200μM), while MitC concentration was kept constant at 0μg/ml, 0.01μg/ml, 0.1μg/ml or 0.25μg/ml or (B) MitC (0.01μg/ml, 0.05μg/ml, 0.1μg/ml 0.2μg/ml, 0.25μg/ml, 0.5μg/ml and 1μg/ml) while the DTPA concentration was kept constant at 0μM, 6μM, 25μM or 100μM. Bacteria were subsequently analyzed by FACS for GFP-signal intensity. Cultures of S. Tm pPcib (multi-copy gfp-reporter) were grown in LB with either increasing concentrations of (C) DTPA (6μM, 12μM, 25μM, 50μM, 100μM and 200μM), while MitC concentration was kept constant at 0.25μg/ml or (D) MitC (0.01μg/ml, 0.05μg/ml, 0.1μg/ml 0.2μg/ml, 0.25μg/ml, 0.5μg/ml and 1μg/ml) while the DTPA concentration was kept constant at 100μM. Bacteria were analyzed by FACS for GFP-signal intensity. S. Tmwt lacking the reporter was used as negative control and for calculating the fraction (%) of GFP+ bacteria (green). (TIF) [file pone.0144647.s001.tif]

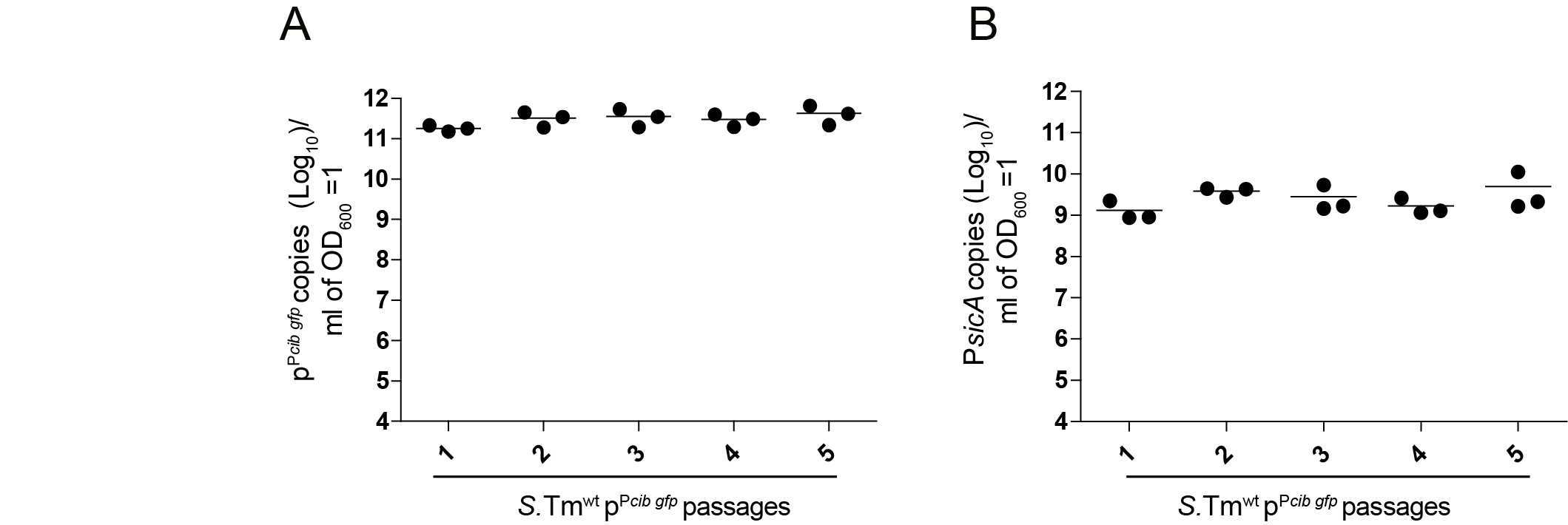

Supplement: S2 Fig — S. Tmwt harboring pPcib gfp was cultured for five consecutive passages (1–5) in 10ml LB. Briefly, a sample of 1ml for an OD600 of 0.4 was taken and used to set up a subculture in 10ml LB liquid media (no antibiotics). This subculture was incubated until OD600 = 2–3. Four more passages were carried out in a similar fashion. From each passage (1–5), samples were taken (1ml from 1 OD600) and total DNA was extracted for quantitative PCR analysis. (A) Copy number of pPcib gfp per ml culture (OD600 of 1) as determined by absolute quantification. (B) Genome copy number as determined by absolute quantification of PsicA copies per ml culture (OD600 of 1). Data were analyzed by 1-way ANOVA. No significant differences were determined between passages. pPcib gfp copy number in S. Tmwt in the 5 consecutive passages (mean±SD) as calculated from data shown in A and B: passage 1: 169±91; passage 2: 84±18; passage 3: 147±57; passage 4: 198±100; passage 5: 127±67. (TIF) [file pone.0144647.s002.tif]

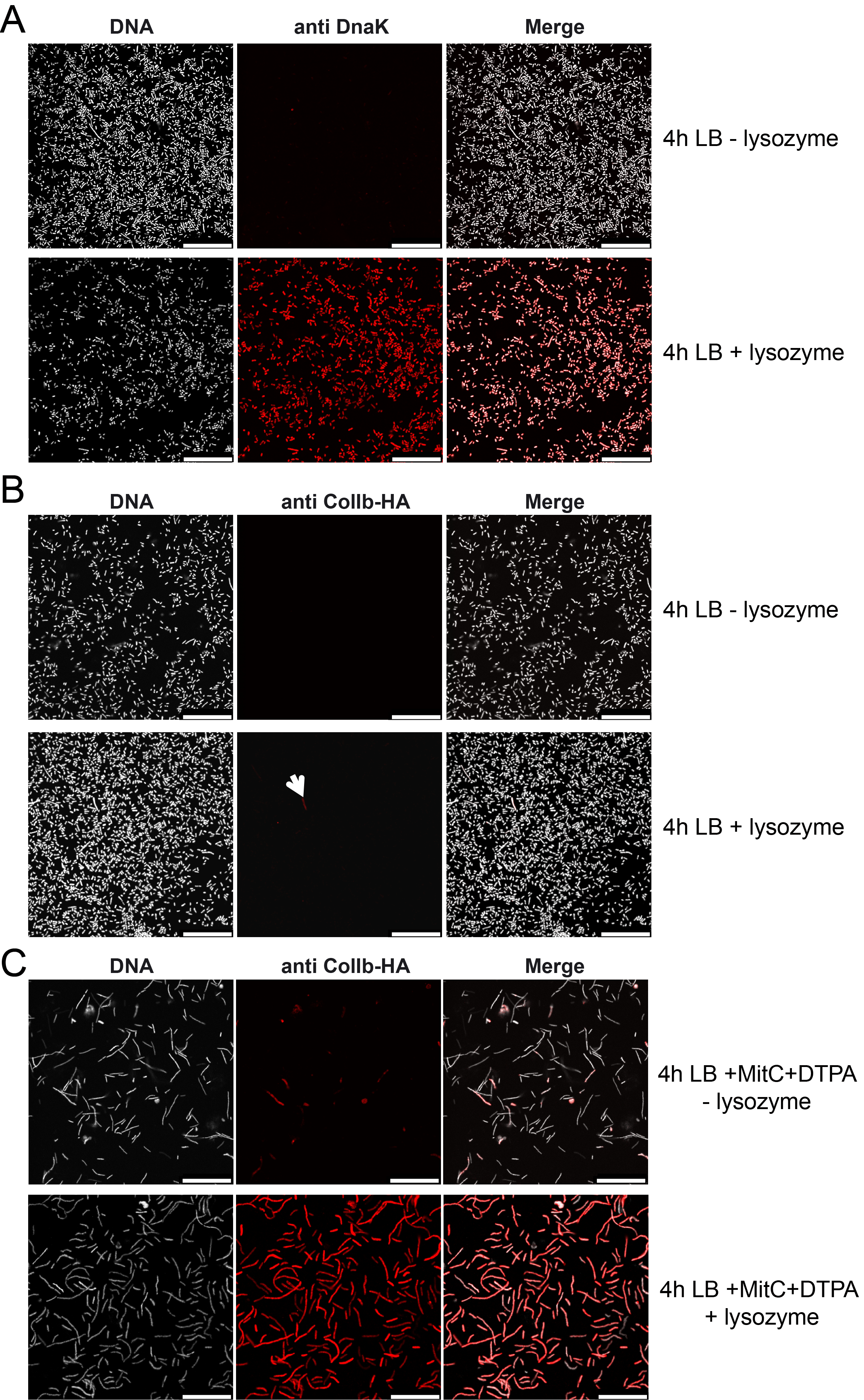

Supplement: S3 Fig — S. Tm p2cib-HA was grown in LB for 12h and 4h in LB or for 4h supplemented with MitC, DTPA or both. The efficiency of lysozyme permeabilization was validated by staining the cytosolic, constitutively expressed protein DnaK in lysozyme-treated (lower panel) and non-treated samples (upper panel) (A). ColIb-HA was detected within a small fraction of S. Tm grown in LB after lysozyme treatment (arrow) but not in untreated samples (upper panel) (B). ColIb-HA was detected in S. Tm grown in LB MitC+DTPA after lysozyme treatment (lower panel) and also in a small fraction of cells without lysozyme treatment (upper panel) (C). Scale bar 25μm. (TIF) [file pone.0144647.s003.tif]

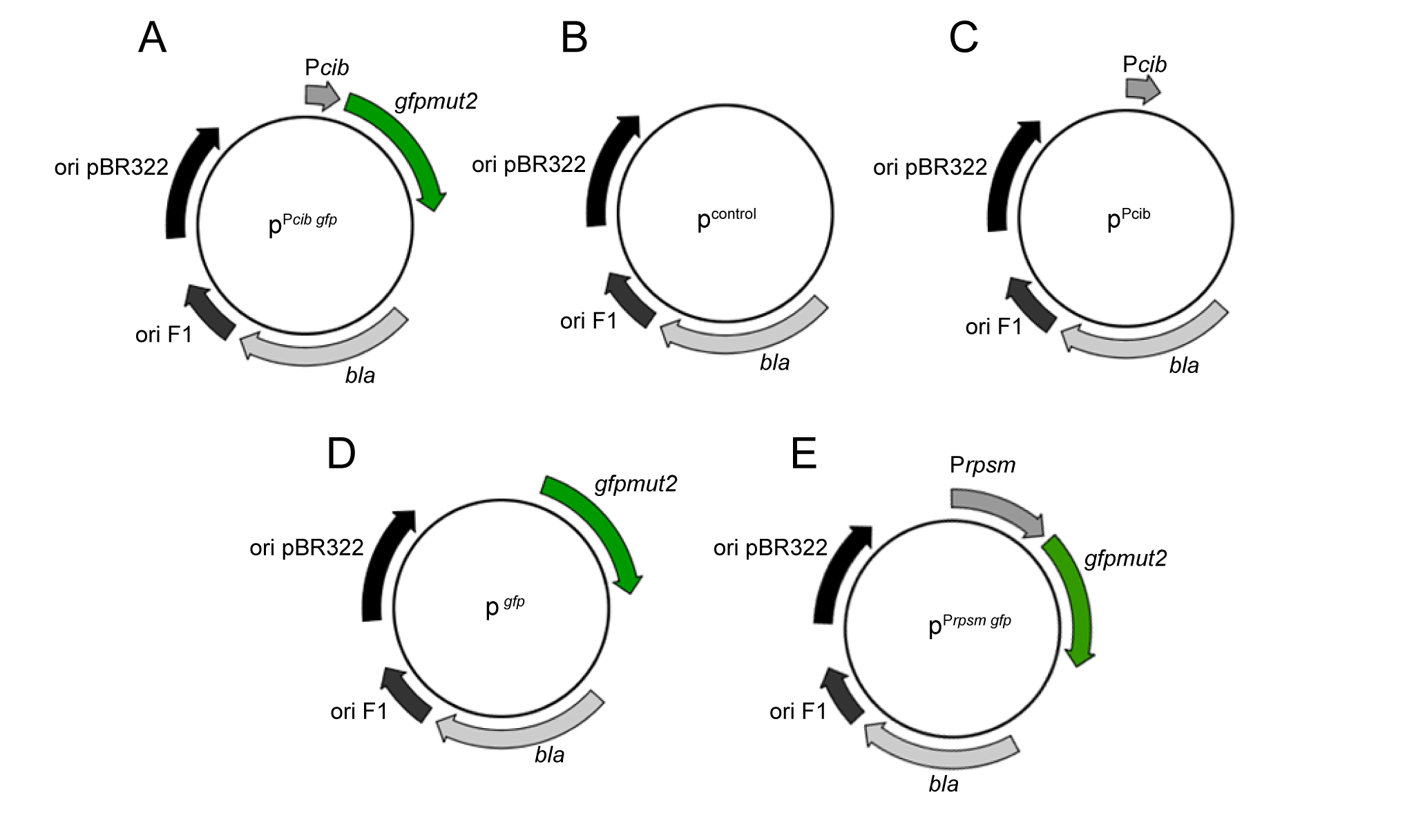

Supplement: S4 Fig — Plasmid maps of (A) pPcib gfp (multicopy reporter); (B) pcontrol (only plasmid backbone) (C) pPcib (only promoter of cib); (D) pgfp (only gfp); (E) pPrpsm gfp (constitutive promoter) are shown. (TIF) [file pone.0144647.s004.tif]

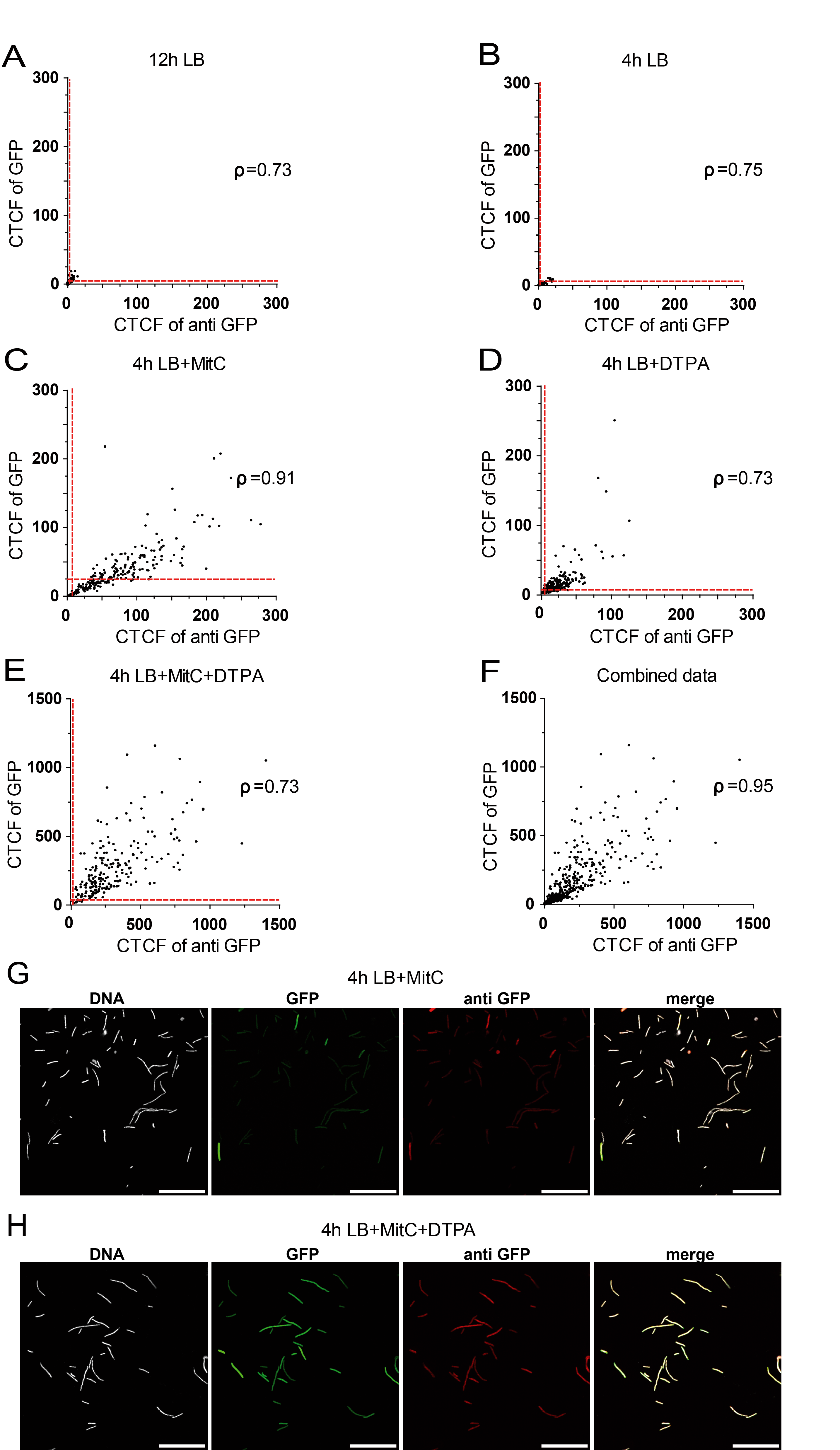

Supplement: S5 Fig — S. Tmwt pPcib gfp was grown for 12h (A) and 4h (B) in LB or for 4h supplemented with MitC (C), DTPA (D) or both (E). Bacteria were fixed, lysozyme permeabilized and stained with a GFP-specific antiserum and a Dylight549-conjugated secondary antibody and analyzed by fluorescence microscopy followed by image analysis. Corrected total cell fluorescence (CTCF) was calculated and GFP-fluorescence of individual bacteria was correlated to Dylight549 fluorescence ([ρ] Spearman-rank correlation coefficient). A combination of all data is shown in (F). Red line: detection limit. Examples for IF-microscopy are shown in (G) and (H). Scale bar 25μm. (TIF) [file pone.0144647.s005.tif]
