# Supplementary material for: Reporters for Single-Cell Analysis of Colicin Ib Expression in Salmonella enterica Serovar Typhimurium
Source: PLoS One. 2015 Dec 10;10(12):e0144647. doi: 10.1371/journal.pone.0144647 (PMC4675545; doi:10.1371/journal.pone.0144647)
Supplement: S1 Table — (DOCX) [file pone.0144647.s006.docx]

**Table S1: Primers used in this study**

| **Designation** | **Sequence (5´-3´)** |
| --- | --- |
| NotI-SFGFP-for | ATAGCGGCCGCATGCGCAAAGGCGAAGAACTG |
| XhoI-SFGFP-rev | ATACTCGAGTTATTATTTATACAGTTCATCCATG |
| SFGFP_cib_fwd | TATACGTAAGCAGTTAATTCATTTGTTTTCCTCAGAGGATGAAGGAGATACCGAATGCGCAAAGGCGAAGAACTGTTTAC |
| SFGFP_cib_rev | TGTGATTATGTTATCACGCACCGGTACACGATAACAATAAAGGAGAAAACAGCGTGTAGGCTGGAGCTGCTTC |
| Check up_SFGFP_fwd | GTGGCTCAGTTCTGTAACT |
| Check up_SFGFP/RFP _rev | CATGCTGGAGTTCTTCGCC |
| Colicin‑HA‑fwd | GTTAATGATAAGTTTATTGAGCAGGTCAATAAACTTATTGGTATCTATCCGTATGATGTTCCTGA |
| Colicin‑HA‑rev | ACTTAATTTTTATATTCTGCAAAGCCCTTTCAGGAAAATGAATATGAATATCCTCCTTAG |
| Col-ÜE-XbaI | CCCTCTAGACCTCAGAGGATGAAGGAGATAC |
| Col-ÜE-XhoI | CCCCTCGAGGATGTAAACGTGACACAGCT |
| check up_pSJB16 | AGGTTTTCACCGTCATCA |
| Ampli1_Fwd | CAGAGGATGAAGGAGATAC |
| Ampli1_Rev | CTCCTTTACGCATGAATAC |
| PsicA_2_fwd | GGC AAA GAC GTT ATT CAG CC |
| PsicA_2_rev | GCCCAATGAATACATCGCTAC |
